# The Proteolytic Activity of Neutrophil-Derived Serine Proteases Bound to the Cell Surface Arming Lung Epithelial Cells for Viral Defense

**DOI:** 10.3390/molecules29184449

**Published:** 2024-09-19

**Authors:** Akmaral Assylbekova, Maiya Allayarova, Moldir Konysbekova, Amanbek Bekturgan, Aiya Makhanova, Samantha Brown, Norbert Grzegorzek, Hubert Kalbacher, Ruslan Kalendar, Timo Burster

**Affiliations:** 1 Department of Biology, School of Sciences and Humanities, Nazarbayev University, Kabanbay Batyr Ave. 53, Astana 010000, Kazakhstanmoldir.konysbekova@nu.edu.kz (M.K.); amanbek.bekturgan@nu.edu.kz (A.B.); aiya.makhanova@nu.edu.kz (A.M.); 2 Institute for Archaeological Sciences, Department of Geosciences, University of Tübingen, 72076 Tübingen, Germany; 3 Mass Spectrometry Facility, Organic Chemistry, Eberhard Karls University Tübingen, 72076 Tübingen, Germany; 4 Institute of Clinical Anatomy and Cell Analysis, University Hospital Tübingen, Eberhard Karls University Tübingen, Österbergstraße 3, 72074 Tübingen, Germany; 5 Center for Life Sciences, National Laboratory Astana, Nazarbayev University, Kabanbay Batyr Ave. 53, Astana 010000, Kazakhstan; ruslan.kalendar@nu.edu.kz

**Keywords:** cathepsin G, neutrophil elastase, proteinase 3, MHC I, SARS-CoV-2, furin, TMPRSS2, protease-catalyzed hydrolysis

## Abstract

The collaboration between cellular proteases and host cells is pivotal in mounting an effective innate immune defense. Of particular interest is the synergistic interaction between cathepsin G (CatG) and neutrophil elastase (NE), which are proteases secreted by activated neutrophils, and the human alveolar basal epithelial cell line (A549) and the human lung epithelial-like cell line (H1299), because of the potential implications for viral infection. Our study aimed to investigate the binding capacity of CatG and NE on the surface of A549 and H1299 cells through preincubation with purified CatG and NE; thereby, the proteolytic activity could be detected using activity-based probes. Both CatG and NE were capable of binding to the cell surface and exhibited proteolytic activity, leading to increased cell surface levels of MHC I molecules, which is crucial for displaying the endogenous antigenic repertoire. In addition, CatG cleaved the S2′ site of the SARS-CoV-2 spike protein at two specific sites (_815_RS_816_ and _817_FI_818_) as well as NE (_813_SK_814_ and _818_IE_819_), which potentially leads to the destruction of the fusion peptide. Additionally, furin required the presence of Ca^2+^ ions for the distinct cleavage site necessary to generate the fusion peptide. Overall, the findings suggest that CatG and NE can fortify target cells against viral entry, underscoring the potential significance of cell surface proteases in protecting against viral invasion.

## 1. Introduction

Upon infection, the immune system orchestrates a complex and highly coordinated response to neutralize invading pathogens. This response is initiated by the innate immune system, where neutrophils play a pivotal role by selectively infiltrating the site of infection and releasing granules containing serine proteases, including neutrophil elastase (NE), cathepsin G (CatG), protease 3 (PR3), and neutrophil serine protease 4 (NSP4), collectively classified as neutrophil serine proteases (NSPs) [1]. Under physiological conditions, on the other hand, continuous and unwanted protease-catalyzed degradation is prevented by serine protease inhibitors (serpins) to avoid cell or tissue damage [2]. In addition to its involvement in antigen processing, the role of CatG in triggering major histocompatibility complex I (MHC I) molecules has been investigated. When exogenous CatG is introduced, this protease leads to an increase in the expression of cell surface MHC I molecules on peripheral blood mononuclear cells (PBMCs), the human acute monocytic leukemia cell line (THP-1), and human glioblastoma cells, which are further upregulated by lactoferrin-enhanced CatG activity, specifically for a B cell line [3]. Upregulated levels of MHC I molecules might result in the display of the target cell’s intracellular antigen status, which can be monitored by CD8^+^ T cells [4]. Indeed, it has been shown that the uptake of enzymatically active NE increases cell surface levels of MHC I and antigen presentation in breast cancer [5] as well as lung cancer cells [6].

Viruses, particularly respiratory viruses like SARS-CoV-2, rely on host cell surface receptors and proteases for entry into target cells. SARS-CoV-2 entry into the host cell is mediated by the Spike protein (S protein), which consists of the following two functional subunits: the S1 subunit for binding to the host cell receptor angiotensin-converting enzyme 2 (ACE2) and the upstream S2 subunit (more precisely, the S2′ site) for fusion with the host cell membrane. Two locations need to be proteolytically cleaved to prepare the S protein for merging with the host cell; the S1/S2 interface can be digested by furin at _685_RS_686_ to separate S1 from the S2 subunit, whereas the remaining S1 subunit is non-covalently connected with the S2 subunit. Remarkably, this process occurs in infected producer cells before virions are released to the extracellular space [7,8,9]. The cleavage of the S2′ site at _815_RS_816_, controlled by the transmembrane protease serine subtype 2 (TMPRSS2) located on the cell surface, creates the formation of the hydrophobic fusion peptide, which is crucial for SARS-CoV-2 to fuse with the host cell membrane [10]. Alternatively, the virus enters the target cell through a TMPRSS2-independent pathway, known as clathrin-mediated endocytosis, where the endosome undergoes gradual step-by-step acidification, which is crucial for the proteolytic activity of the cysteine protease cathepsin L (CatL). At a lower pH, CatL cleaves the S2′ site, enabling SARS-CoV-2 to fuse with the endosomal membrane and release its contents into the cytosol [11,12]. The viral genome does not encode the necessary proteases for priming the S protein; therefore, the host’s proteolytic enzymes are “hijacked” for entry.

Recently, a study demonstrated that NSPs, such as CatG, NE, and, to a lesser extent, PR3 can degrade the S protein of SARS-CoV-2 as an innate defense, consequently reducing viral infection and supporting viral clearance [13]. We found that CatG and NE can bind to the cell surface of A549 and H1299 cells, are proteolytically active, provoke the upregulation of MHC I molecules, and potentially destroy the fusion peptide, which impacts innate defense as well as antigen presentation.

## 2. Results

### 2.1. CatG and NE Bind to the Cell Surface of A549 and H1299 Cells in a Proteolytically Active Conformation

Our aim was to elucidate whether CatG and NE, a protease secreted by activated neutrophils into the extracellular space [14], are capable of binding in a proteolytic manner to the surface of the human alveolar basal epithelial cell line (A549) or human epithelial-like, non-small cell lung carcinoma cell line derived from the lymph node (H1299). To do so, we preincubated these cells with purified CatG or NE and then detected the proteolytic activity of both proteases using the following activity-based probes (ABPs). MARS116-FAM or VPV-FAM probes contain a phosphonate warhead that reacts with the nucleophilic oxygen atom of the active site serine residue in the catalytic center of CatG or NE, respectively, resulting in a covalent bond between the phosphonate and the serine side chain. This complex can be visualized, and the proteolytic activity can be determined by different approaches, including fluorescence microscopy [15,16,17].

**Figure 1:**
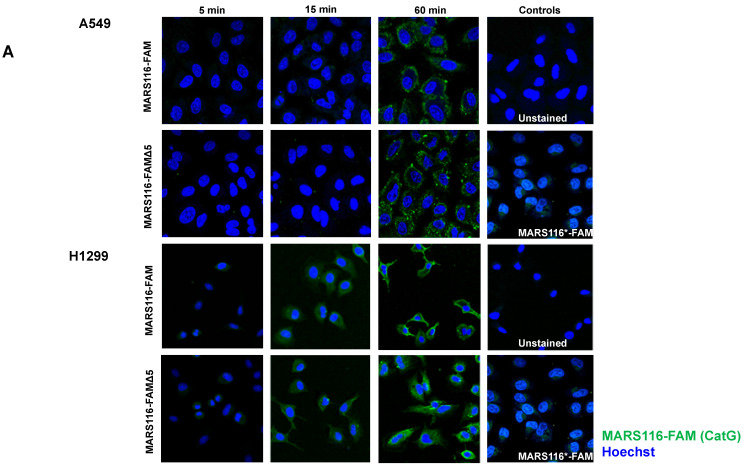

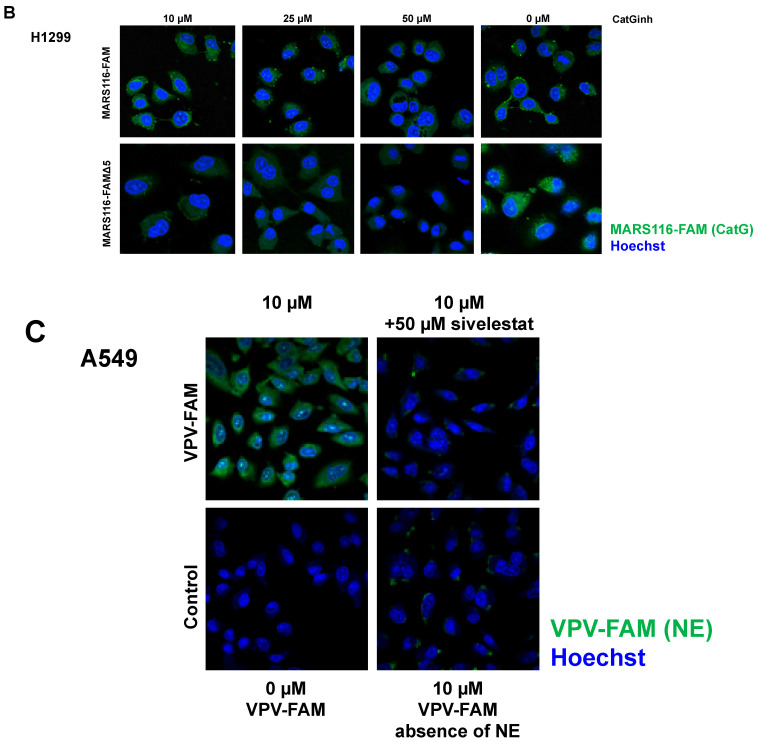
Catalytically active CatG and NE bind to the cell surface of A549 and H1299 cells and are internalized. (**A**) Confocal microscopy images illustrate A549 and H1299 cells incubated with CatG [10 µg/mL] complexed with MARS116-FAM or MARS116-FAMΔ5 [0.5 µM] at various time points (5, 10, and 60 min) at RT. The data presented are representative of three independent experiments (n = 3). (**B**) CatG [10 µg/mL] was preincubated with the CatG inhibitor (10, 25, and 50 µM), followed by adding MARS116-FAM [0.5 µM]. Catalytically active CatG was determined in H1299 cells by confocal microscopy; n = 3. (**C**) A549 cells were incubated with NE [10 µg/mL] complexed with VPV-FAM [10 µM]. The data represent findings from two separate, independent experiments.

**Figure 2:**
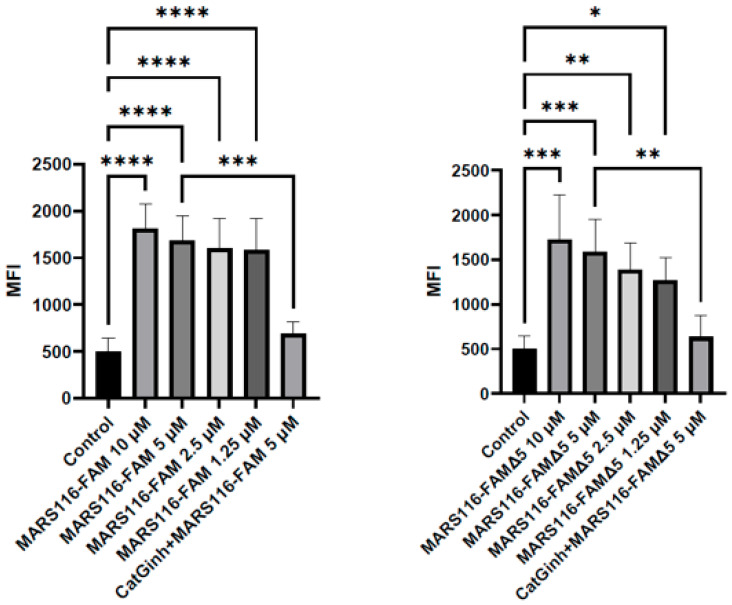
Detection of active CatG at the cell surface applying MARS116-FAM and MARS116-FAMΔ5. A549 cells were incubated with exogenous CatG [10 μg/mL] and were stained with increasing concentrations of MARS116-FAM or MARS116-FAMΔ5 (1.25–10 μM) in the presence or absence of the CatG inhibitor. Propidium iodide (PI) was added before cell collection by flow cytometry. While the *y*-axis represents median fluorescence intensity (MFI) in arbitrary units (AUs), the *x*-axis denotes the concentrations of MARS116-FAM or MARS116-FAMΔ5. Error bars depict standard deviation, and significance was calculated by an unpaired one-way ANOVA and Sidak post hoc test (*p* < 0.05 = *, *p* < 0.01 = **, *p* < 0.001 = ***, and *p* < 0.0001 = ****). Four independent experiments, n = 4.

In an initial experiment, the impact of nutritional conditions (with or without FBS) on CatG activity was investigated at both the in vitro and cellular levels. Specifically, the turnover of the colorimetric substrate by CatG was tested and revealed that in the presence of FBS, the catalytic performance of CatG was impaired (Supplementary Data S1, [18]). Confocal microscopy images showed a substantial increase in fluorescence intensity in cells subjected to starvation (medium without FBS) in contrast to cells under normal conditions (as evidenced in the Supplementary Data S2), indicating higher activity of catalytically active CatG to nutrient deprivation. Furthermore, to minimize steric hindrance during binding to the catalytic center of CatG on the cell surface, MARS116-FAM∆5, which has a shorter spacer because the truncation of one CH_2_ group changes the conformation of the probe, was initially tested using an SDS-PAGE approach and found that both ABPs detected the proteolytic activity of CatG in a similar fashion (Supplementary Data S3). Proteolytically active CatG was sensed on the surface of A549 and H1299 cells (Figure 1A). Over time, the intensity of fluorescence observed on the cell surface showed a consistent increase, indicating that the binding of active CatG to the cell surface is time-dependent. In addition, an ABP lacking the reactive phosphonate “warhead” (MARS-116*-FAM) was included to exclude nonspecific binding. To validate that the observed activity was specifically due to CatG, the CatG inhibitor was included in the assay, resulting in a reduction in activity that correlated with the concentration of the inhibitor (Figure 1B). Similar to CatG, NE bound to the cell surface of A549 cells, but NE was internalized into the cells to a greater extent once bound to the cell surface compared with CatG (Figure 1C). The comparison of the median fluorescence intensity (MFI) of both MARS116 ABPs using flow cytometry revealed the presence of active CatG on the cell surface (Figure 2 and Supplementary Data S4) and NE (Supplementary Data S5). Thus, the observation suggests that CatG and NE can bind to the cell surface of A549 and H1299 cells and are proteolytically active.

### 2.2. CatG and NE Provoke the Upregulation of MHC I on A549, H1299, and Jurkat Cells

**Figure 3:**
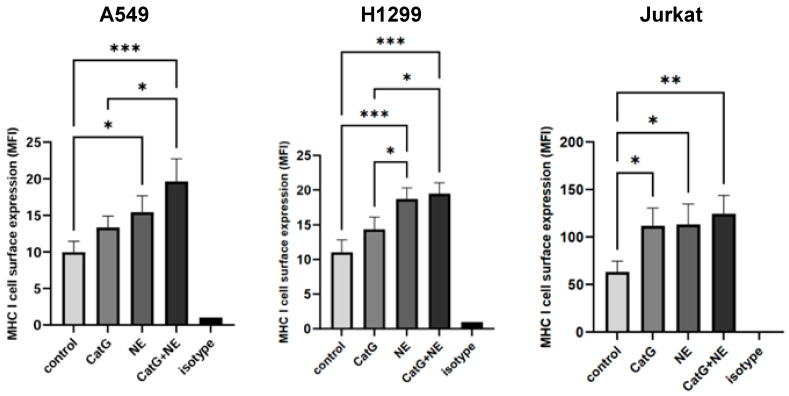
MHC I cell surface expression increases after treatment with proteases. A549, H1299, and Jurkat cells were incubated with CatG [10 μg/mL], NE [10 μg/mL], or both for 6 h in PBS, and the MHC I cell surface expression was analyzed using flow cytometry. Data were normalized to isotype control and considered significant at *p* < 0.05 (*), *p* < 0.01 (**), or *p* < 0.001 (***) by using an unpaired one-way ANOVA and Sidak post hoc test. n = 3.

In the first set of experiments, we tested the potential effects of CatG or NE on the viability of cells. To verify this, A549 cells were incubated with the indicated proteases, and the regulation of apoptosis markers was determined by using the Human Apoptosis Array Kit. Our findings showed that neither CatG nor NE induced apoptosis in the A549 or H1299 cells, as shown in Supplementary Data S6 and S7. In a previous publication, we demonstrated that CatG provoked the upregulation of MHC I molecules on immune cells and glioblastoma cells [3]. Furthermore, Chawla et al. reported a similar effect with NE on breast cancer cells, where levels of MHC I molecules were increased on the cell surface in a concentration- and time-dependent form [5]. Peters and colleagues also showed that the serine protease NE enhanced the presentation of MHC I immunogenic peptides on lung cancer cells [6]. Based on published data, A549, H1299, or Jurkat cells were incubated with either CatG, NE, or a combination of both for a duration of 6 h. Strikingly, only NE significantly induced the upregulation of MHC I molecules on A549 cells and H1299, with the combination of CatG and NE having a slightly additive effect compared with Jurkat cells, where CatG and NE upregulated MHC I molecules, as depicted in Figure 3 (Supplementary Data S8).

### 2.3. The S2′ Site Is a Substrate for CatG and NE

**Figure 4:**
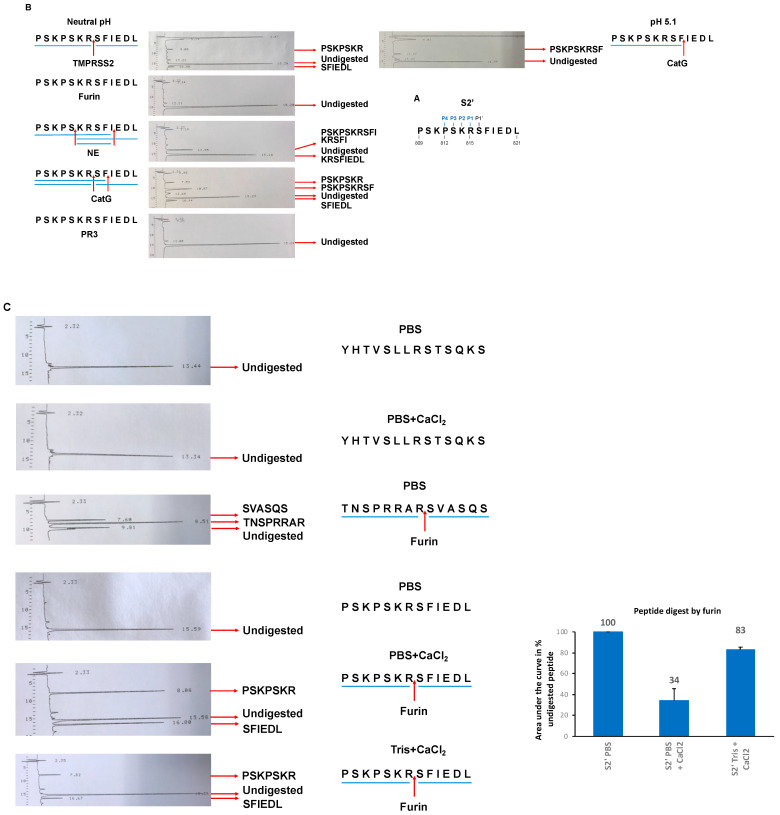
Summary of the proteolytic cleavage sites of proteases at the S2′ site. (**A**) Amino acid alignment of the S2′ region of the SARS-CoV-2 S protein. (**B**) S2′-peptides were incubated with TMPRSS2, furin, NE, CatG, and PR3 for 2 h at 37 °C. The hydrolysis of the peptide bonds is summarized in a digestion map (blue bars denote the fragments, and red arrows indicate the cleavage sites). Three independent experiments, n = 3. (**C**) Peptides were incubated with furin in the presence or absence of additional Ca^2+^ ions, with a CaCl_2_ final concentration of 1.2 mM (left panel). Quantification (right panel). n = 3.

Recent findings have indicated that CatG, NE, and, to a lesser extent, PR3 degraded the S protein of SARS-CoV-2, resulting in reduced viral infection [13]. Therefore, we wondered whether these proteases could generate or destroy the fusion peptide, which is crucial for SARS-CoV-2 to enter the target cell. We speculated that potential future mutations would develop on the S protein to eliminate the cleavage site for NSPs, which would allow the S protein to evade destruction. On the other hand, the S2′ site has a conserved amino acid sequence. In order to determine if the S2′ site is a substrate for several serine proteases, a peptide was synthesized spanning the S2′ peptide _809_PSKPSKRSFIEDL_821_ (S2′-peptide, Figure 4A) and was incubated with the serine proteases TMPRSS2 (control), NE, CatG, furin, or PR3 under neutral conditions, and CatG also at pH 5.1. The resulting digestion pattern was resolved by HPLC and analyzed by using mass spectrometry. The fragments and the cleavage sites were summarized in a digestion map, as shown in Figure 4B (Supplementary Data S9 and S10). TMPRSS2 hydrolyzed the peptide bond between _815_RS_816_ as expected [19], while NE digested _813_SK_814_ and _818_IE_819_. Strikingly, CatG catalyzed the hydrolysis of the peptide bond between _815_RS_816_ and _817_FI_818_ at a neutral pH, compared with pH 5.1 only at _817_FI_818_, but furin and PR3 did not digest the S2′-peptide.

To further investigate why furin does not hydrolyze the peptide bond of the S2′-peptide, digestion was repeated with peptides spanning the S1/S2 interface of the S protein of SARS-CoV-1 (_660_YHTVSLLRSTSQKS_673_), the polybasic sequence of SARS-CoV-2 (_678_TNSPRRARSVASQS_691_) [20], and the S2′-peptide with and without adding CaCl_2_. Indeed, furin hydrolyzed the peptide bond between _815_RS_816_ of S2′-peptide depending on the Ca^2+^ concentration and the buffer used in the assay, which was most efficient in the PBS buffer (Figure 4C and Supplementary Data S11). This contrasts the _660_YHTVSLLRSTSQKS_673_ peptide, which was not proteolyzed by furin. The peptide _678_TNSPRRARSVASQS_691_ was used as a positive control [20]. Thus, the Ca^2+^ environment dictates the cleavage capacity of furin, which might explain why the S1/S2 interface is primed intracellularly by furin during productive infection in contrast to the S2′ site.

## 3. Discussion

The interaction between cell surface proteases and the environment plays a crucial role in understanding the cellular function and response to inflammation. One such interaction might involve the attachment of NSPs to cells, leading to significant implications possibly for immune defense. We found that CatG and NE bind to the cell surface of A549 and H1299 cells and were proteolytically active. Both NSPs were associated with an increased expression of MHC I molecules, which is indicative of a potential impact on the MHC I-mediated immune response.

A recent study discussed that the outcome of SARS-CoV-2 infection differs among individuals, potentially because of varying levels of proteases responsible for degrading the virus [21], and NSPs degrade the S protein of SARS-CoV-2, consequently reducing viral infection [13]. On the other hand, it has been suggested that amino acid substitutions during SARS-CoV-2 mutation could lead to the creation of new cleavage sites for NSPs, potentially increasing viral entry [20,22,23]. Interestingly, in the infected producer cells, the S1 and S2 subunits are already separated by furin-catalyzed hydrolysis (S1/S2 interface at _685_RS_686_), while the remaining S1 subunit is non-covalently connected with the S2 subunit and is sufficient to bind to the entrance receptor [8]. This suggests that the S1/S2 interface may not need to be cleaved prior to binding to ACE2, and it is crucial that the conserved S2′ site is precisely digested, generating the fusion peptide. Furthermore, CatG was shown to bind to immune cells (summarized in [4]) and can be internalized in B cells to the lysosomal compartment [24]. NE, PR3, and most likely CatG bind to the cell surface of endothelial cells and are subsequently internalized, where these proteases are enzymatically active [25] and the S protein contains multiple cleavage sites that are susceptible to degradation by NSPs [13]. Moreover, NE and PR3 were found to cleave the viral F-protein of the human respiratory syncytial virus, which is responsible for viral adhesion and fusion with the target cell [26], and based on our findings (Figure 1 and Figure 2), this leads us to assume that cell surface NSPs bind to the cell surface of lung cells in a proteolytically active conformation to arm such cells to fight against a harsh viral environment. Additionally, this conclusion provides an explanation of why CatG is present on the cell surface of B cells, CD4^+^, CD8^+^ T cells, dendritic cells, monocytes, neutrophils, and natural killer cells, indicating an innate immune (cellular) defense strategy against viral infection.

Upon entering the cell via clathrin pit-mediated endocytosis [27], NE supports the stabilization and increase in cell surface MHC I molecules by decreasing the turnover of MHC I molecules in the cell [5], thereby enhancing immunogenic antigen presentation via MHC I [6]. In the case of CatG, it has been proposed that CatG upregulates MHC I via the cell surface protease-activated receptor 1 (PAR1) by increasing the recycling process of MHC I back to the cell surface [3,4]. Strikingly, the levels of MHC I molecules on Jurkat cells are increased further by CatG and NE compared with the levels observed on A549 or H1299 cells. This suggests that CatG and NE have a pronounced effect on the expression of MHC I molecules on Jurkat cells, indicating potential differences in antigen presentation compared with the other cell types and suggesting that immune cells in transit may require heightened surveillance for infection to prevent the dissemination of the virus.

In a possible scenario when an infection occurs, neutrophils enter the site of inflammation and release NSPs. As a result, NSPs bind to the surface of lung cells. During viral engagement, NSPs might degrade the S protein to prevent the virus from entering the host cell. This binding might lead to the upregulation of MHC I molecules, presenting antigen peptides to indicate the intracellular cell status, and in the case of infection, viral-derived antigenic peptides are presented on MHC I molecules for CD8^+^ T cell inspection. Consequently, the activation of CD8^+^ T cells induces apoptosis in the infected cell to prevent productive infection and avoid the release of new virions. On the other hand, mutations in the S protein may particularly occur on the non-essential entrance site, potentially leading to amino acid substitutions at the S2′ site. This eliminates destructive cleavage sites since CatG cleaves at two sites (_815_RS_816_ and _817_FI_818_) as well as NE (_813_SK_814_ and _818_IE_819_) and most likely destroys the fusion peptide. One possible mutation is that the _815_RS_816_ cleavage site remains for CatG to generate the fusion peptide independently of the Ca^2+^ concentration on the cell surface environment, which is needed for furin (Figure 4). However, TMPRSS2 catalyzes the hydrolysis of the peptide bond between _815_RS_816_ and facilitates the generation of the fusion peptide [28].

Even though the protective function of the serine protease inhibitor SerpinA1 (alpha1-antitrypsin) in COVID-19 is controversial, in general, SerpinA1 inhibits CatG, NE, PR3, and TMPRSS2, and is known to be typically upregulated in response to managing inflammation and tissue damage, which is important for the immune system to return to homeostasis [29,30]. The effectiveness of SerpinA1 in completely inhibiting CatG under physiological conditions is currently uncertain, as the association rate constant is higher for PR3 and, to a greater extent, for NE. It is more important to determine the regulation of other SerpinA molecules, specifically SerpinA3, which is a more potent CatG inhibitor [31] and was found to be upregulated in critical COVID-19 patients [32]. One scenario could be that the levels of SerpinA3 molecules are decreased during viral infection in some individuals, leading to inadequate inhibition of CatG. This lack of inhibition may, in turn, support viral clearance, particularly in asymptomatic—in contrast to symptomatic—individuals. Interestingly, lactoferrin (LF) has been found to enhance the catalytic activity of CatG through an allosteric mechanism [33], which could be an additional explanation for the role of LF in viral defense. However, the application of LF in clinical trials is controversial. As an example, LF reduced respiratory tract infection [34], but, in contrast, LF did not have any beneficial effects on symptomatic infection [35]. Thus, other factors, such as the upregulation of SerpinA3 in symptomatic patients and long COVID, need to be taken into consideration.

## 4. Materials and Methods

### 4.1. Cell Culture

The following cell lines were used in this study: A549 cells (CCL-185, American Type Culture Collection, ATCC, Manassas, VA, USA), an adenocarcinoma human alveolar basal epithelial cell line; NCI-H1299 cells (H1299, CRL-5803, American Type Culture Collection, ATCC, Manassas, VA, USA), a human epithelial-like, non-small cell lung carcinoma cell line derived from the lymph node; and Jurkat E6-1 cells (Jurkat, human T cell lymphoblasts, ARP-177, BEI Resources; NIAID, NIH, Bethesda, MD, USA [36]). The cells were cultured in RPMI-1640 medium (R8755-1L, Lot No. SLBF1516V, Sigma-Aldrich, St. Louis, MO, USA) supplemented with 10% Fetal Bovine Serum (FBS heat-inactivated at 56 °C for 30 min, Lot No. 2631713RP, Gibco, Grand Island, NY, USA) and 1% Penicillin–Streptomycin (pen-strep, Lot No. 210496, Gibco, Grand Island, NY, USA).

A549 and H1299 cells were propagated until they reached 80–90% confluency. The medium was aspirated, and the cells were washed with prewarmed PBS, pH 7.4 (Lot No. RNBL5991, Sigma-Aldrich, St. Louis, MO, USA). To detach the cells, 2 mL of 0.5% Trypsin-EDTA (Lot No. 2537762, Gibco, Grand Island, NY, USA) was added to the cells and incubated for 3 min in the CO_2_ incubator at 37 °C or until cell detachment was observed under the microscope. The detached cells were then resuspended in 8 mL of complete medium (RPMI-1640, 10% FBS, and 1% pen-strep) and transferred to a 15 mL tube. The cell suspension was centrifuged for 5 min at 200xg at RT. The medium was changed on day 2 or 3. Jurkat cells were maintained and subcultured in the same complete growth medium (RPMI-1640, 10%FBS, and 1% pen-strep). The passage was performed every 3 days by inoculating 0.5 × 10^6^ cells/mL.

### 4.2. Confocal Microscopy

Cells were initially seeded on glass-bottom µ-slides (Ibidi GmbH, Gräfelfing, Germany) in a complete medium, consisting of RPMI-1640, 10% FBS, and 1% pen-strep, for 24 h. After the first day of incubation, the media of the samples were changed to serum-free conditions for an additional 24 h. Generally, the ABPs MARS116-FAM [0.5 µM], MARS116*-FAM without phosphonate [0.5 µM], or MARS116-FAMΔ5 [0.5 µM], which has a shorter spacer due to the truncation of one CH_2_ group, were applied for CatG [10 µg/mL]. VPV-FAM [0.5 µM] was used for NE [10 µg/mL] detection [15,16,17]. The ABPs were kindly provided by Professor Dr. Marcin Sienczyk and Professor Dr. Renata Grzywa (Division of Medicinal Chemistry and Microbiology, Faculty of Chemistry, Wroclaw University of Technology, Wroclaw, Poland). The protease and ABP were incubated in a serum-free medium for 40 min at room temperature (RT) and protected from light. The CatG inhibitor I (CatGinh, Calbiochem Research Biochemicals, Schwalbach, Germany) was used as a control. Afterward, the protease–ABP complex was added to the cells for the indicated time points. The cells were then fixed in ice-cold 99% methanol for 5 min and washed with PBS pH 7.2 twice. Next, the cells were subjected to staining with Hoechst dye (Ready Probes Reagent ™, Thermo Fisher Scientific, Carlsbad, CA, USA) 5 min prior to confocal microscopy analysis using ZEISS LSM 780 (Carl Zeiss AG, Jena, Germany). Hoechst was detected by the DAPI channel (405 nm), and ABPs were analyzed using the FITC filter (488 nm). Channels were captured sequentially. All images were acquired using ZEN microscopy software (ZEN 2011 SP7 FP3, version 14.0.23.201, Carl Zeiss AG, Jena, Germany) and further processed in FIJI software (doi:10.1038/nmeth.2019, 4 July 2023).

### 4.3. Detection of Cell Surface CatG Activity by Flow Cytometry

A solution of CatG with a final concentration of 10 µg/mL was mixed with CatGinh (Calbiochem Research Biochemicals, Schwalbach, Germany) at a final concentration of 50 µM in PBS pH 7.4 and incubated for 15 min at RT. The A549 or H1299 cells were adjusted to 5 × 10^6^ cells/mL. The CatG-CatGinh complex or CatG was then added to the cells. Increasing concentrations of MARS116-FAM or MARS116-FAMΔ5 were added to the cells for 40 min (the cells were covered to protect them from light). The cells were then washed twice with FACS buffer (PBS pH 7.4 and 1% FBS). Subsequently, the cells were resuspended with 400 µL of FACS buffer and stained with propidium iodide (PI) at a final concentration of 0.5 µg/mL. The cells were collected and analyzed by using an Attune Nxt Flow Cytometer (Thermo Fisher Scientific, Waltham, MA, USA) and FlowJo™ v10.8.1 Software (BD Life Sciences, Ashland, OR, USA). The control samples with CatGinh were used to identify the specificity of each ABP. The gating strategy was based on the forward vs. side scatter (FSC/SSC) profile. The cells of interest were obtained by gating the cell population based on size and granularity (FSC vs. SSC) to exclude debris. Single cells were identified by using forward scatter height (FSC-H) compared with forward scatter area (FSC-A) for double cell exclusion, and viable cells were identified by gating on the single cell population, which was negative for PI.

### 4.4. Analysis of Cell Surface MHC I by Flow Cytometry

A549, H1299, and Jurkat cells were seeded into 24-well plates in complete medium (RPMI-1640, 10% FBS, and 1% pen-strep) one day before treatment. On the day of the experiment, the cells were incubated with CatG [10 μg/mL], NE [10 μg/mL], or a combination of both in PBS pH 7.4 for 6 h. Before staining, the A549 and H1299 cells were trypsinized, and the pellets of all 3 cell types were washed once with 5 mL of PBS pH 7.4. The cell pellets were transferred to 1.5 mL tubes and washed again with FACS buffer (250 μL PBS pH 7.4 with 1% FBS). The cells were stained with an anti-human HLA-ABC monoclonal antibody (W6/32) conjugated with APC (Lot No. 4289568, eBioscience by Thermo Fisher Scientific, Waltham, MA, USA) or with the isotype control mouse IgG2a kappa (eBM2a) conjugated to APC (Lot No. 4289571, eBioscience, Waltham, MA, USA) in 100 μL of FACS buffer and incubated for 30 min on ice. Following incubation, the cells were washed with FACS buffer and resuspended with 250 μL of FACS buffer. The samples were immediately analyzed using the flow cytometer (Attune Nxt Flow Cytometer, Thermo Fisher Scientific, Waltham, MA, USA). Median fluorescence intensity (MFI) in the RL1-A channel was used for statistical analysis.

### 4.5. Peptide Hydrolysis by the Proteolytic Activity of Proteases

The peptides were ordered at GeneCust (Boynes, France) and further purified by using a reversed-phase high-performance liquid chromatography (HPLC) column (C18, 250 × 8 column, Dr. Maisch GmbH, Ammerbuch-Entringen, Germany). Afterward, the peptides were lyophilized and dissolved in PBS pH 7.4 (final concentration 10 mg/mL). The exact molecular weight was determined by matrix-assisted laser desorption/ionization-time-of-flight mass spectrometry (Autoflex mass spectrometry from Bruker Daltonics, Bremen, Germany, or Autoflex Max mass spectrometry from Bruker Daltonics, Bremen, Germany) utilizing a matrix containing 2,5-Dihydroxybenzoic acid (DHB) from Bruker Daltonics, Bremen, Germany.

Human NE (4 μg/mL, neutrophil-derived human NE, PN: 16-14-051200, Lot No. EH 2020-03, Athens Research and Technology, Athens, GA, USA), human CatG (4 μg/mL, neutrophil-derived CatG, PN: 16-14-030107, Lot No. CG 2017-01, Athens Research and Technology, Athens, GA, USA), 4 μg/mL recombinant human furin (4 μL furin, containing 5 mM CaCl_2_ based on the company’s production, was added to 94 μL PBS pH 7.4, with a final concentration of 0.2 mM; furin Cat. No. 450-47, Lot No. 1011516, Peprotech, Cranbury, NJ, USA) with or without the addition of CaCl_2_ to a final concentration of 1.2 mM, or recombinant human TMPRSS2 (4 μg/mL, Cat. No. CSB-YP023924HU, Batch No. DA0471a3g001, BIOZOL, CUSABIO Technology, LLC, TX, USA) was incubated with 200 μg/mL peptide in PBS pH 7.4 (or in the case of TMPRSS2, Tris pH 7.8, 7.7 mM Tris/HCl and 150 mM NaCl) for 2 h at 37 °C. The digestion pattern was determined by using a Reprosil 100, 250 × 2 mm, C18 with a 5 μm particle diameter column (Dr. Maisch GmbH, Ammerbuch-Entringen, Germany), the separation was achieved by a linear acetonitrile gradient, and the eluted peptides (fragments) were identified at 214 nm (UV vis detector L-4200, Merck-Hitachi, Darmstadt, Germany, and the Chromato-Integrator D-2500, Merck-Hitachi, Darmstadt, Germany). Autoflex mass spectrometry from Bruker Daltonics (Bremen, Germany) or Autoflex Max mass spectrometry from Bruker Daltonics (Bremen, Germany) was applied to reveal the exact molecular mass, which was manually linked to the peptide prediction tool as indicated in the ExPASy FindPept tool (https://web.expasy.org/findpept, 27 July 2023, Swiss Institute of Bioinformatics, Lausanne, Switzerland).

### 4.6. Statistical Analysis

The data were analyzed with the commercially available software GraphPad Prism 10.2.3 (San Diego, CA, USA), and the data were quantified and expressed as intensity. The +/− standard error of the mean (S.E.M.) is shown with significant differences at *p* < 0.05 (*), *p* < 0.01 (**), *p* < 0.001 (***), or *p* < 0.0001 (****), as indicated in the figures and figure legends accompanied by an explanation of which statistical significance test was employed.

## 5. Conclusions

CatG and NE bond to the cell surface of lung cells, exhibit proteolytic activity, increase the levels of MHC I molecules essential for presenting the internal antigenic repertoire, and most likely degrade the fusion peptide. These findings indicate that CatG and NE are capable of strengthening target cells against viral invasion.

## Data Availability

Data is contained within the article or Supplementary Materials.

## Supplementary Materials

The following supporting information can be downloaded at https://www.mdpi.com/article/10.3390/molecules29184449/s1: Data S1: CatG activity was determined using MARS116-FAM and compared to MARS116-FAMΔ5; Data S2: Substrate turnover of Suc-VPF-pNA [200 μM] by CatG [2.5 μg/mL] using different conditions; Data S3: Analysis of CatG activity under normal nutritional conditions and starvation; Data S4: Titration of ABPs; Data S5: NE binding on the cell surface of A549 cells; Data S6: The apoptosis profiler array (CatG); Data 7: The apoptosis profiler array (NE); Data S8: MHC I expression on the cell surface of Jurkat cells, treated with CatG under different conditions; Data S9: Mass spectrometry data, Data S10: Control experiment to resolve if PR3 was active; Data S11: Mass spectrometry data (furin).
