# Supplementary material for: The Proteolytic Activity of Neutrophil-Derived Serine Proteases Bound to the Cell Surface Arming Lung Epithelial Cells for Viral Defense
Source: Molecules. 2024 Sep 19;29(18):4449. doi: 10.3390/molecules29184449 (PMC11434079; doi:10.3390/molecules29184449)
Supplement: Supplementary file 1 [file molecules-29-04449-s001.zip › molecules-3160505-supplementary.pdf]

Supplementary data S1

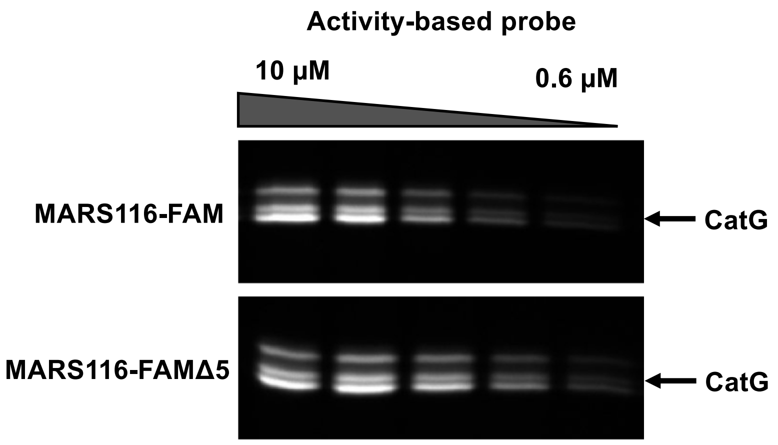

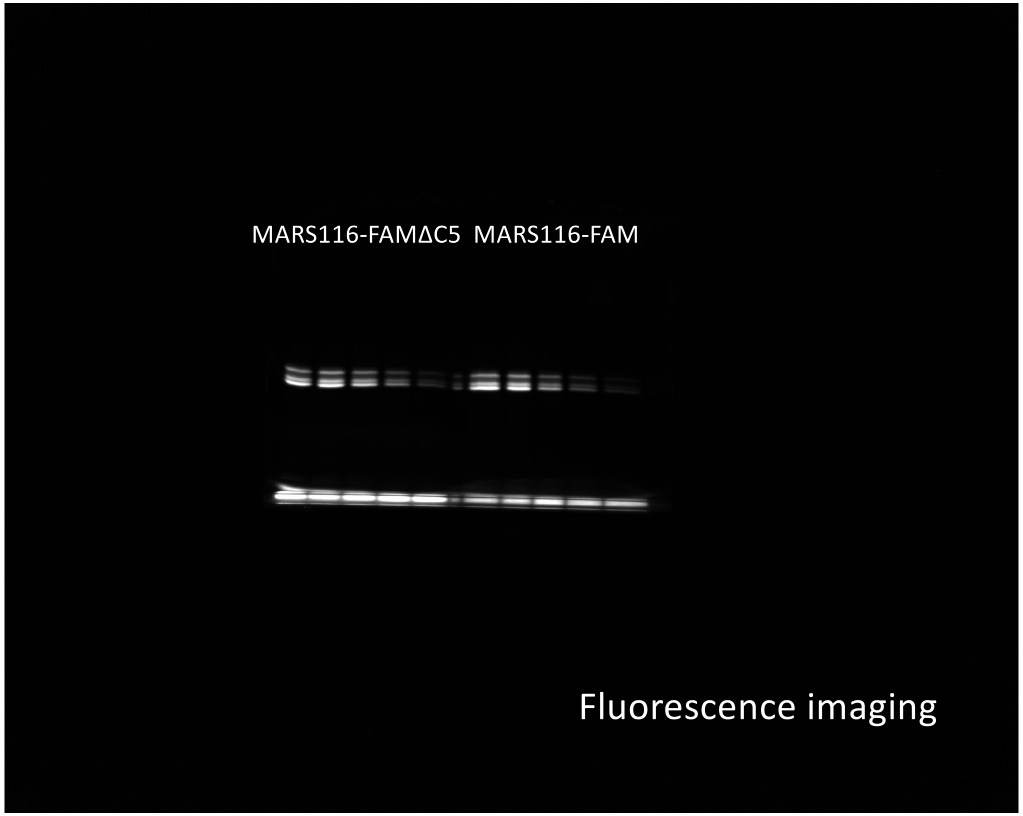

Raw data (tiff) from ChemicDoc Imager

CatG activity was determined using MARS116-FAM and compared to MARS116-FAMΔ5. A representative SDS-PAGE gel image showing the titration of ABP, showing the fluorescence intensity of CatG-ABP [40 µg/mL]. Increasing concentrations of MARS116-FAM or MARS116-FAMΔ5 was as followed 0.625, 1.25, 2.5, 5, and 10 µM final concentration and were incubated with CatG in PBS pH 7.4 for 45 min at RT. Images were taken using ChemiDoc imager (ChemiDoc MP and Image Lab Touch Software, 2.4.0.03, BioRad, Hercules, CA, USA) and analyzed in Fiji software (Wayne Rasband and contributors, National Institutes of Health, USA, doi:10.1038/nmeth.2019). n=3.

Supplementary data S2

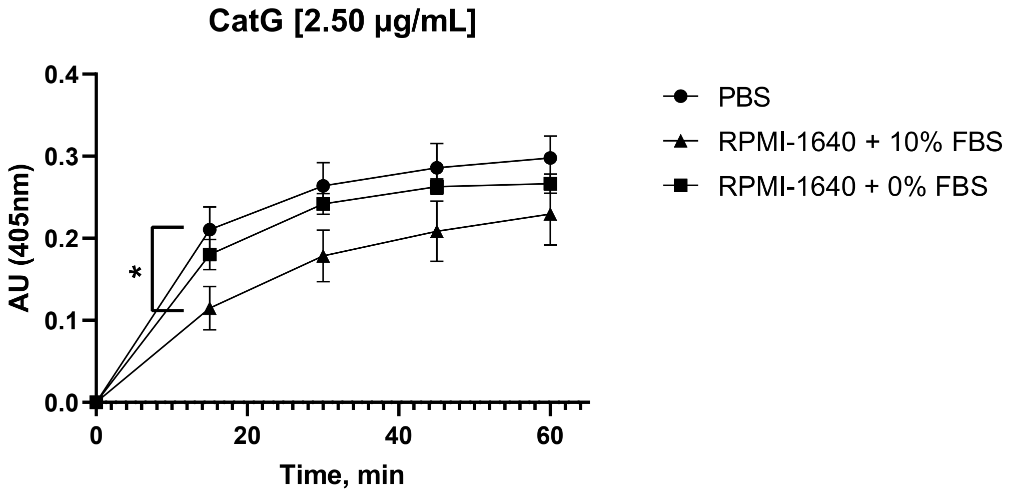

Substrate turnover of Suc-VPF-pNA [200 μM] by CatG [2.5 µg/mL] using different conditions. PBS; complete RPMI-1640 (10% FBS); serum-free RPMI-1640 (0% FBS). Measurements were performed at 405 nm using a 96-well plate reader (Varioskan Flash Multimode Reader, Thermo Fisher Scientific, Waltham, MA, USA) in duplicates. Significant differences at $p < 0.05$ (*), 2 independent experiments.

Supplementary data S3

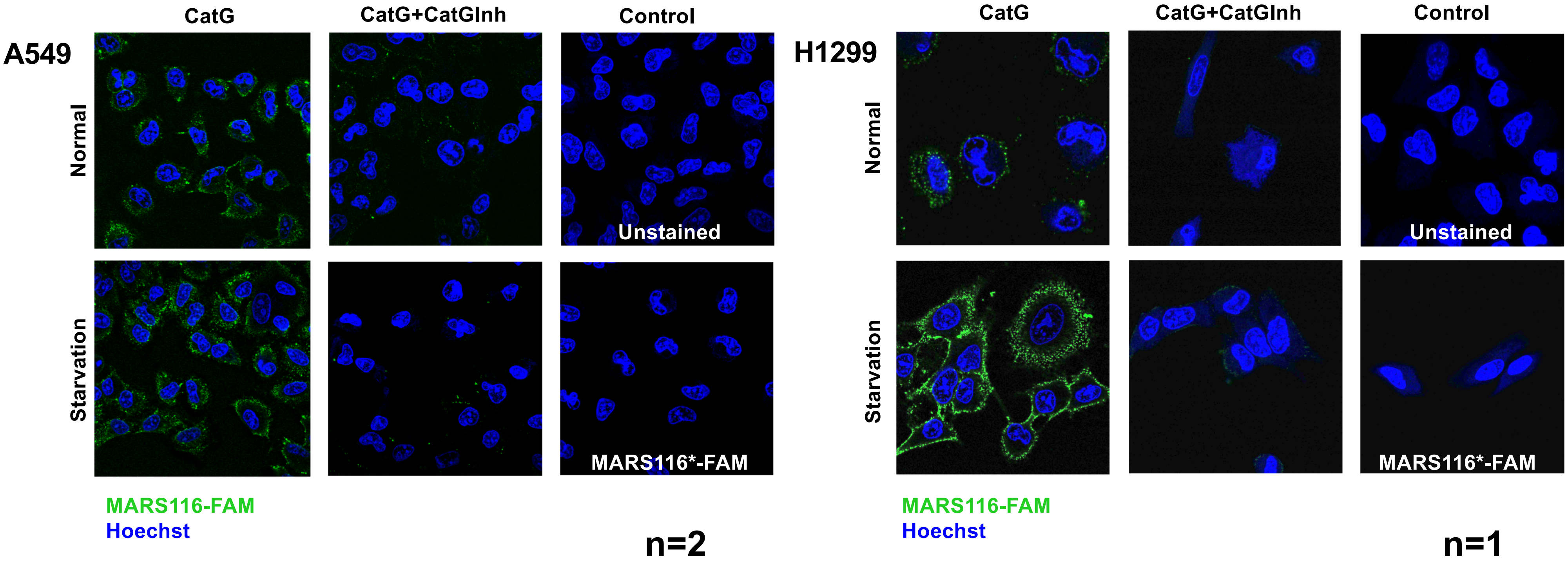

Analysis of CatG activity under normal nutritional conditions and starvation. A549 (left panel) and H1299 cells (right panel). The image shows the cellular localization of exogenously added CatG (green) under normal nutritional conditions (upper panel) or during starvation (lower panel). CatG, at a final concentration of 10 µg/mL, was incubated with or without CatGinh and MARS116-FAM [0.5 µM] for 40 min before being added to the cells. MARS116-FAM represents an additional control without phosphonate (“warhead”); n=2.

Supplementary data S4

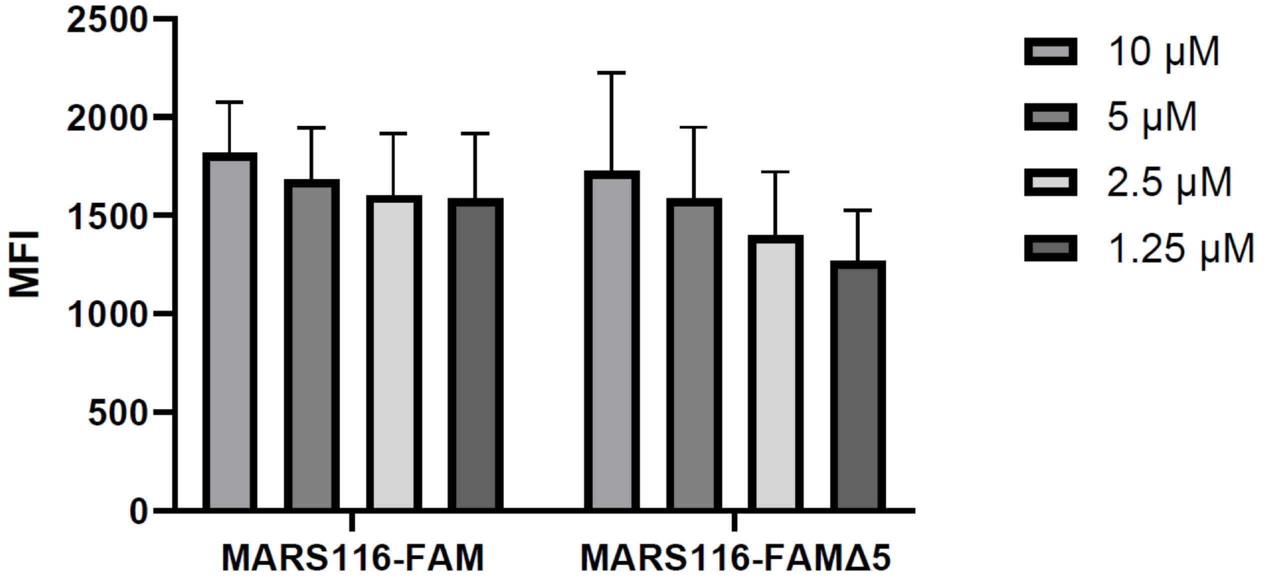

Titration of ABPs to determine the ABP concentration for flow cytometry. A549 cells were treated with CatG [10 µg/ml] and incubated with MARS116-FAM or MARS116-FAMΔ5 for 40 min. CatG activity on the cell surface was analyzed using flow cytometry (Attune NxT Flow Cytometer, Thermo Fisher Scientific, Waltham, MA USA). Error bars depict standard deviation, and significance was determined by using unpaired two-way ANOVA and Sidak post hoc test (not significant). n=4.

Supplementary data S5

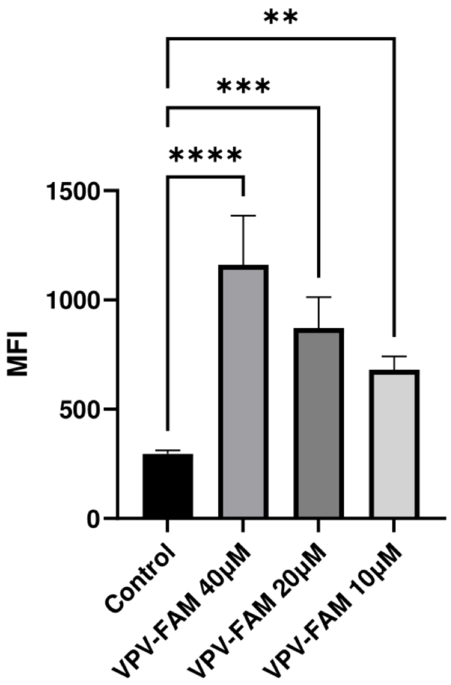

NE binding on the cell surface of A549 cells. NE [10 μg/mL] were preincubated (40 min) with increasing concentrations of VPV-FAM [10-40 μM] and then added to A549 cells for 60 min. Propidium iodide (PI) was added before cell collection by flow cytometry. While the y-axis represents median fluorescence intensity (MFI) in arbitrary units (AU), the x-axis denotes the concentrations of VPV-FAM. Error bars depict standard deviations, and significance considered at $p < 0.01$ (**), $p < 0.001$ (***), or $p < 0.0001$ (****) by using the unpaired one-way ANOVA and Sidak post hoc test. The experiments were done in duplicates, two independent experiments, n=2.

Supplementary data S6

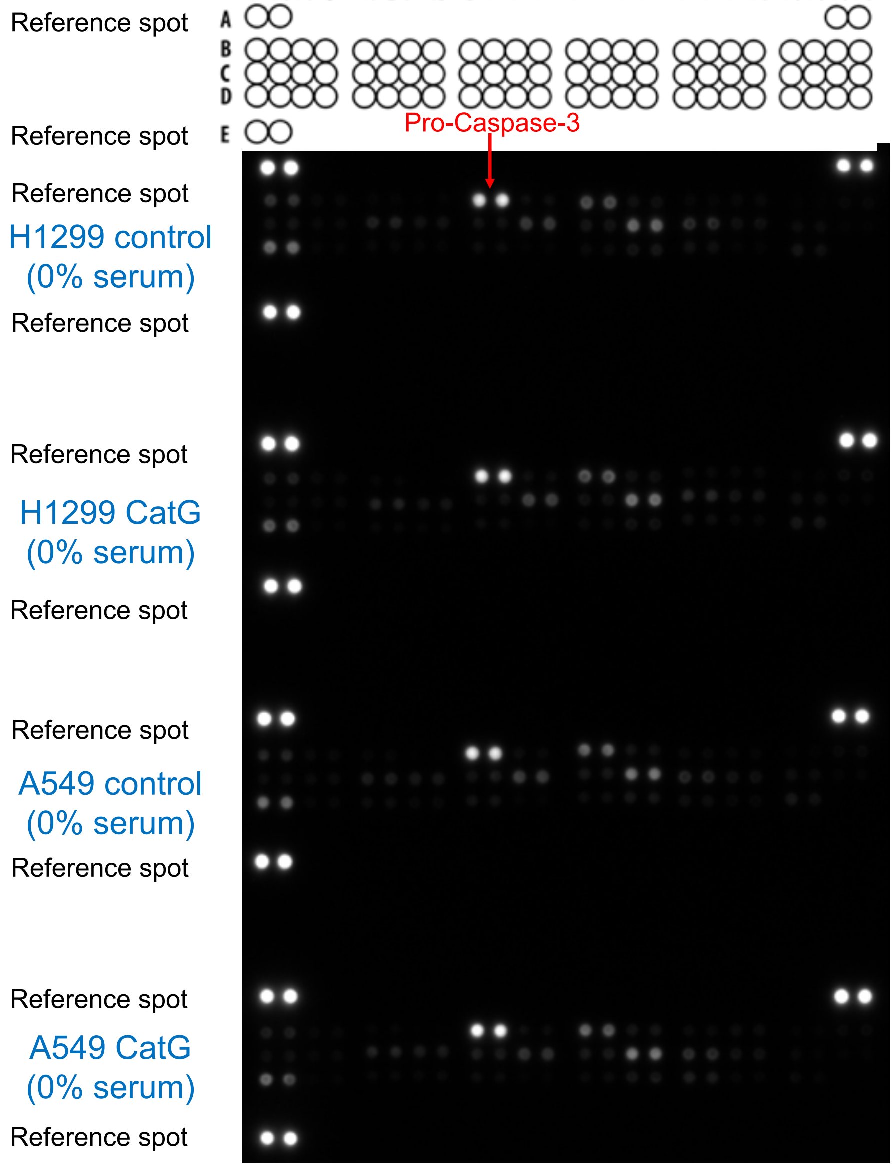

Coordinate	Target/Control	Coodinate	Target/Control
A1, A2	Reference Spots	C13, C14	HO-2/HMOX2
A23, A24	Reference Spots	C15, C16	HSP27
B1, B2	Bad	C17, C18	HSP60
B3, B4	Bax	C19, C20	HSP70
B5, B6	Bcl-2	C21, C22	HTRA2/Omi
B7, B8	Bcl-x	C23, C24	Livin
B9, B10	Pro-Caspase-3	D1, D2	PON2
B11, B12	Cleaved Caspase-3	D3, D4	p21/CIP1/CDKN1A
B13, B14	Catalase	D5, D6	p27/Kip1
B15, B16	cIAP-1	D7, D8	Phospho-p53 (S15)
B17, B18	cIAP-2	D9, D10	Phospho-p53 (S46)
B19, B20	Claspin	D11, D12	Phospho-p53 (S392)
B21, B22	Clusterin	D13, D14	Phospho-Rad17 (S635)
B23, B24	Cytochrome c	D15, D16	SMAC/Diablo
C1, C2	TRAIL R1/DR4	D17, D18	Survivin
C3, C4	TRAIL R2/DR5	D19, D20	TNF RI/TNFRSF1A
C5, C6	FADD	D21, D22	XIAP
C7, C8	Fas/TNFRSF6/CD95	D23, D24	PBS (Negative Control)
C9, C10	HIF-1α	E1, E2	Reference Spots
C11, C12	HO-1/HMOX1/HSP32		

The apoptosis profiler array (CatG). H1299 and A549 cells were treated with CatG [10 μg/mL] and incubated for 16 h. The apoptosis profiler array (Proteome Profiler™ Human Apoptosis Array Kit, Catalog #: ARY009, R&D Systems, Inc., Minneapolis, MN, USA) was applied to detect different apoptosis markers in cell lysate (150 μg) according to the manufacturer's protocol. The membranes were imagined by the chemiluminescence detector (ChemiDoc MP and Image Lab Touch Software, 2.4.0.03, BioRad, Hercules, CA, USA). n=2

Supplementary data S7

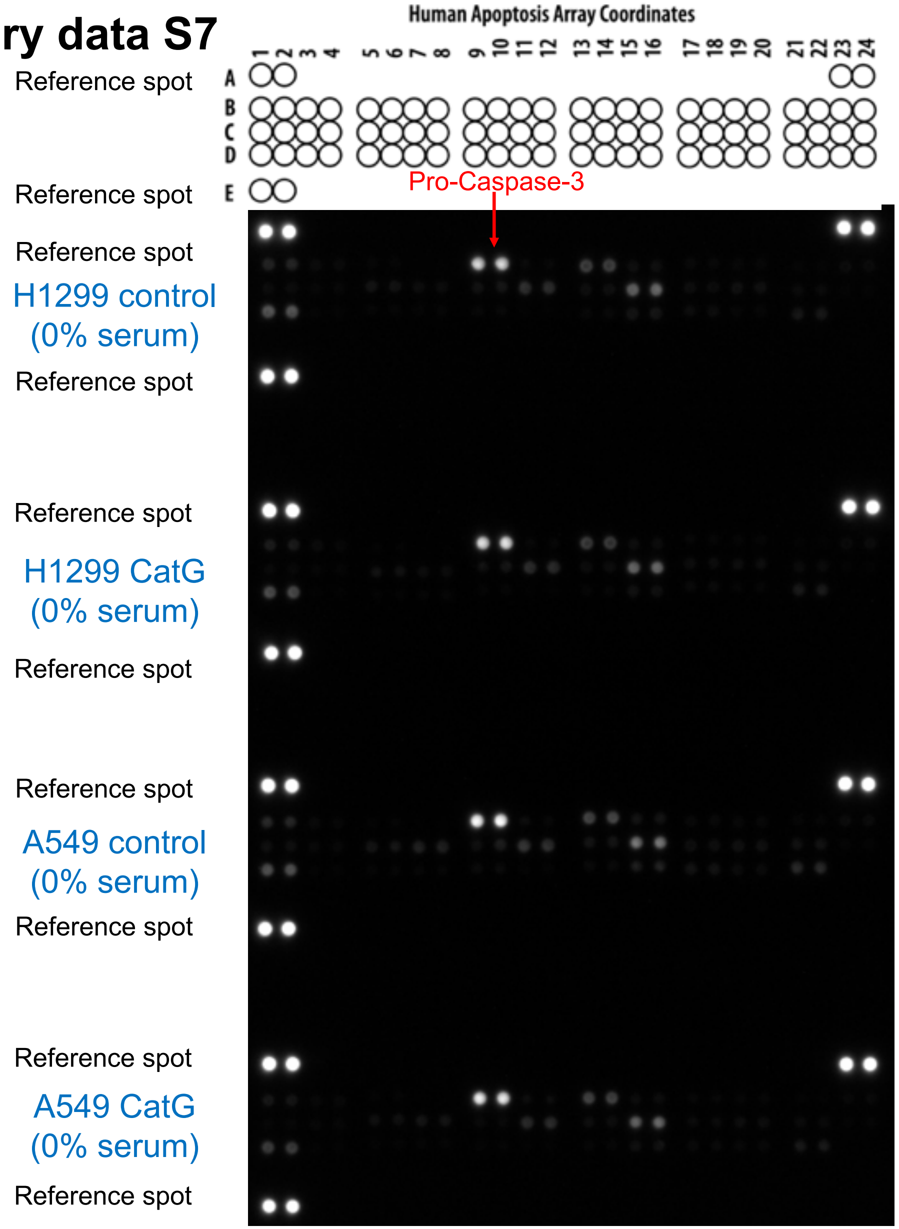

Coordinate	Target/Control	Coodinate	Target/Control
A1, A2	Reference Spots	C13, C14	HO-2/HMOX2
A23, A24	Reference Spots	C15, C16	HSP27
B1, B2	Bad	C17, C18	HSP60
B3, B4	Bax	C19, C20	HSP70
B5, B6	Bcl-2	C21, C22	HTRA2/Omi
B7, B8	Bcl-x	C23, C24	Livin
B9, B10	Pro-Caspase-3	D1, D2	PON2
B11, B12	Cleaved Caspase-3	D3, D4	p21/CIP1/CDKN1A
B13, B14	Catalase	D5, D6	p27/Kip1
B15, B16	cIAP-1	D7, D8	Phospho-p53 (S15)
B17, B18	cIAP-2	D9, D10	Phospho-p53 (S46)
B19, B20	Claspin	D11, D12	Phospho-p53 (S392)
B21, B22	Clusterin	D13, D14	Phospho-Rad17 (S635)
B23, B24	Cytochrome c	D15, D16	SMAC/Diablo
C1, C2	TRAIL R1/DR4	D17, D18	Survivin
C3, C4	TRAIL R2/DR5	D19, D20	TNF RI/TNFRSF1A
C5, C6	FADD	D21, D22	XIAP
C7, C8	Fas/TNFRSF6/CD95	D23, D24	PBS (Negative Control)
C9, C10	HIF-1α	E1, E2	Reference Spots
C11, C12	HO-1/HMOX1/HSP32		

The apoptosis profiler array (NE). H1299 and A549 cells were treated with NE [10μg/mL] and incubated for 16h. The apoptosis profiler array (Proteome Profiler™ Human Apoptosis Array Kit, Catalog #: ARY009, R&D Systems, Inc., Minneapolis, MN, USA) was applied to detect up to different apoptosis markers in cell lysate (150 μg) according to the manufacturer's protocol. The membranes were imagined by the chemiluminescence detector (ChemiDoc MP and Image Lab Touch Software, 2.4.0.03, BioRad, Hercules, CA, USA). n=2

Supplementary data S8

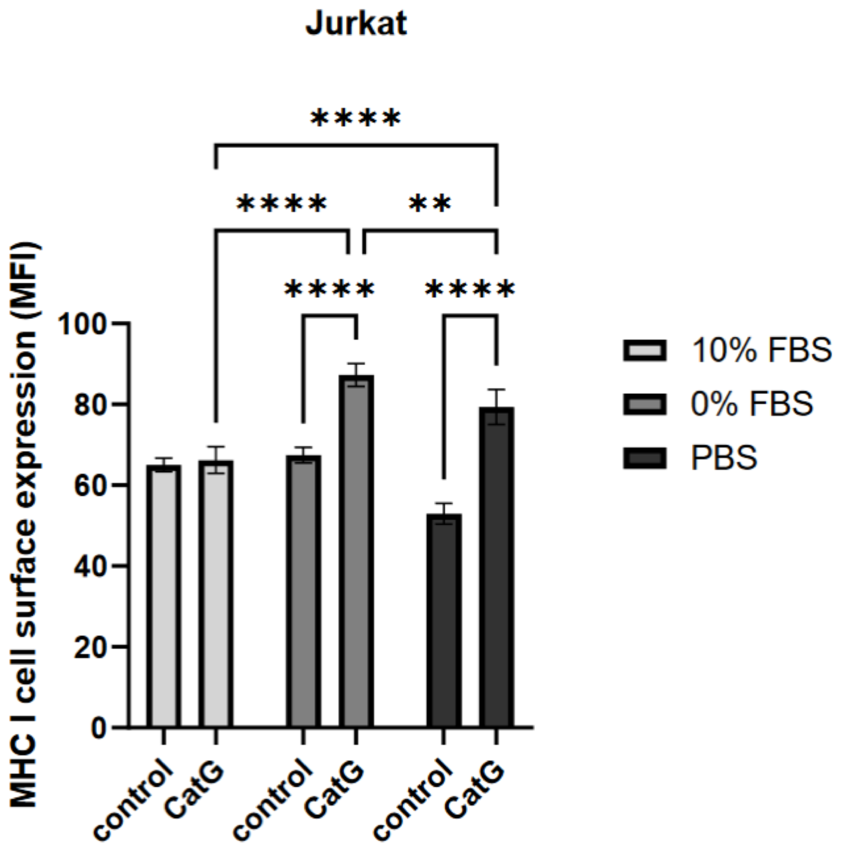

MHC I expression on the cell surface of Jurkat cells. Jurkat cells were treated with CatG [10 µg/mL] under different conditions. RPMI-1640 + 10% FBS, RPMI-1640 + 0% FBS or PBS for 6 h. MHC I cell surface expression was then analyzed using flow cytometry (Attune NxT Flow Cytometer, Thermo Fisher Scientific, Waltham, MA USA). The data were normalized to the isotype control and considered significant at $p < 0.01$ (**), $p < 0.0001$ (****), and not significant at $p > 0.05$ (ns) by using unpaired two-way ANOVA and Tukey's multiple comparisons test. n=4.

Supplementary data S9

Mass spectrometry data.
Summary of the mass spectrometry data. The digestion pattern was analyzed by HPLC and mass spectrometry.

S2': PSKPSKRSFIEDL			
**Protease**	**Mass $[M+H]^+$**	**Expected mass $[M+H]^+$**	**Peptide**
TMPRSS2	723.33	723.36	SFIEDL
Tris buffer	799.44	799.48	PSKPSKR
	1503.15	1503.82	undigested
Furin	1503.66	1503.82	undigested
NE	650.48	650.40	KRSFI
	1007.40	1007.55	KRSFIEDL
	1146.67	1146.66	PSKPSKRSFI
	1503.59	1503.82	undigested
CatG pH 7.4	723.40	723.36	SFIEDL
	799.48	799.48	PSKPSKR
	1033.69	1033.58	PSKPSKRSF
	1504.05	1503.82	undigested
PR3	1503.68	1503.82	undigested
CatG pH 5.1	1033.94	1033.58	PSKPSKRSF
	1504.21	1503.82	undigested

Supplementary data S10

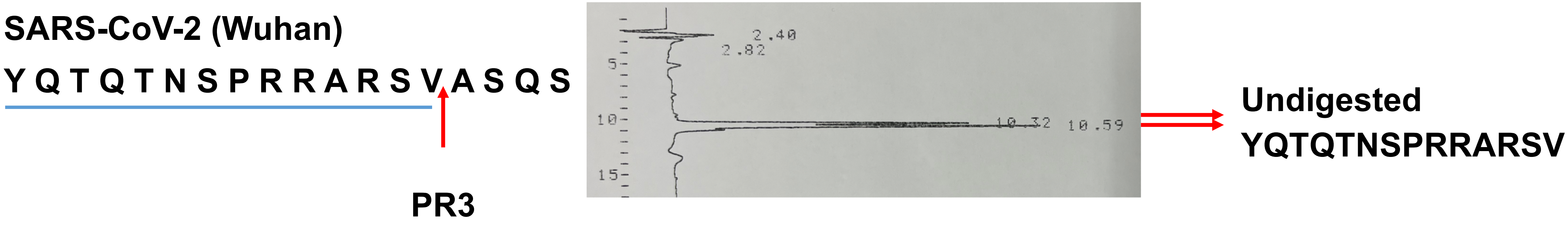

Control experiment to resolve that PR3 is active. The peptide (200 μg/mL) were incubated with 4 μg/mL PR3 for 2 h at 37°C and followed by quantification of the peptide turnover by using HPLC and the cleavage site was determined by mass spectrometry; n=1.

Supplementary data S11

Mass spectrometry data (furin).
Summary of the mass spectrometry data. The digestion pattern was analyzed by HPLC and mass spectrometry.

S2': PSKPSKRSFIEDL

Protease	Mass $[M+H]^+$	Expected mass $[M+H]^+$	Peptide
Furin	1503.47	1503.82	undigested
PBS			
Furin	799.31	799.48	PSKPSKR
PBS	1504.19	1503.82	undigested
1mM $CaCl_2$	723.31	723.36	SFIEDL
Furin	799.48	799.48	PSKPSKR
Tris	1503.97	1503.82	undigested
1mM $CaCl_2$	723.28	723.36	SFIEDL

SARS-CoV-2: TNSPRRARSVASQS

Protease	Mass $[M+H]^+$	Expected mass $[M+H]^+$	Peptide
Furin	578.84	578.28	SVASQS
	957.49	957.53	TNSPRRAR
	1516.30	1516.79	undigested

SARS-CoV-1: YHTVSLLRSTSQKS

Protease	Mass $[M+H]^+$	Expected mass $[M+H]^+$	Peptide
Furin	1605.31	1606.86	undigested
Furin	1605.57	1606.86	undigested
PBS			
1mM $CaCl_2$			
